# Bridging the Gap between Translational and Outcome Research in Cardiovascular Disease

**DOI:** 10.1155/2015/454680

**Published:** 2015-10-27

**Authors:** Giacomo Frati, Umberto Benedetto, Giuseppe Biondi-Zoccai, Sebastiano Sciarretta

**Affiliations:** ^1^Department of Medico-Surgical Sciences and Biotechnologies, Sapienza University of Rome, Corso della Repubblica 79, 04100 Latina, Italy; ^2^Department of AngioCardioNeurology, IRCCS Neuromed, Via Atinense 18, 86077 Pozzilli, Italy; ^3^Oxford Heart Center, Oxford University Hospital, Headley Way, Oxford OX3 9DU, UK; ^4^Eleonora Lorillard Spencer Cenci Foundation, Rome, Italy; ^5^Department of Cell Biology and Molecular Medicine, Rutgers New Jersey Medical School, 185 South Orange Avenue, Newark, NJ 07103, USA

The adjective “translational” stems from Ancient Latin “translatio” (transfero, carry over). Translational research is the branch of scientific research aimed at transferring discoveries obtained in basic research investigations to the clinical context. Accordingly, in the field of biomedical sciences the concept of translational research relates to the transfer of scientific knowledge into practical clinical applications in order to improve the management and treatment of human diseases.

Medicine in general is particularly fascinated by the translational concept, probably for its peculiar applied scopes, and cardiology is not an exception. In this regard, the International Society for Cardiovascular Translational Research (http://www.isctr.org/) was founded in 2007 with the objective to coordinate and guide basic and clinical researchers, regulatory authorities, and the medical industry to improve the process of transferring new scientific evidence into clinical applications, promote translational research and divulgate new scientific results to the scientific community, and develop guidelines for conducting translational research studies. In addition, the ISCTR and the American College of Cardiology (ACC) collaborative efforts began in 2010 involving education on translational science pathways.

The reason behind the interest for translation research in the cardiovascular field is obvious. Although huge advances in the treatment of cardiovascular diseases have been made during the last decades, the morbidity and mortality associated with heart diseases are still too high [1]. In particular, the incidence of heart failure continues to increase with a progressively higher overload for national health systems and health care providers involved in the management of this chronic and highly disabling disease. For all these reasons, it is crucial to develop new therapeutic strategies for the prevention and treatment of cardiac diseases, with a particular focus on heart failure. However, the only way to be successful in this difficult task is to implement the efforts devoted to translational research.

One important goal of cardiovascular translational research is the discovery of new biomarkers that could be useful for the diagnosis, prognostic stratification, and therapeutic management of specific cardiac diseases [2]. This can be achieved by testing whether factors, which were previously found to be associated with specific cardiovascular disease models in experimental studies, are indeed useful for the clinical management of patients affected by these illnesses. These biomarkers could be circulating humoral factors or specific cellular subtypes, or preclinical markers of organ damage. For example, it is now well established that a reduction of flow-mediated artery dilation in human subjects is a preclinical sign of endothelial dysfunction and an independent predictor of adverse cardiovascular events [3].

Another important interest of translational research is the elucidation of the genetic basis of cardiovascular diseases. In fact, the clarification of the genetic predisposition to cardiovascular illnesses can help to identify those subjects who need a different clinical management. Genome-wide association studies are continuously providing new insights into the gene variants associated with cardiovascular sicknesses [4]. However, case-cohort studies or longitudinal investigations are also brilliantly identifying new polymorphisms linked to an increased incidence of cardiovascular diseases or to a worse prognosis. For example, recent studies demonstrated that T2238C atrial natriuretic peptide gene variant is independently associated with an increased incidence of adverse cardiovascular events [5].

A fundamental field in cardiovascular research is also represented by the study of cardioprotection [6]. In fact, the discovery of new therapies reducing the amount of myocardial death during an acute myocardial infarction would help to significantly reduce the incidence of subsequent cardiac dysfunction and heart failure. This would be possible only if the mechanisms regulating cardiomyocyte survival and death during myocardial ischemia are elucidated so that appropriate therapeutic targets can be identified. Unfortunately, in the last decades only few of the therapeutic interventions effective in the reduction of myocardial infarction and cardiac remodeling in animal models of ischemia, ischemia/reperfusion, and chronic myocardial infarction resulted to be effective also in patients experiencing a real heart attack. This may be due to the fact that the animal models of myocardial ischemia and infarction currently employed in basic experimental studies do not accurately mimic the pathophysiology of a human myocardial infarction [7]. Future efforts of researchers working on cardioprotection should be devoted to the development of more relevant models of cardiac diseases.

Finally, a fascinating and promising field of translational research is represented by cardiac regeneration. The possibility for physicians to repair a failing heart with stem cells or applied tissue-engineered myocardial patches still represents a new frontier for the treatment of heart failure [8]. Unfortunately, as in the field of cardioprotection, most of the attempts to effectively translate the highly promising experimental results obtained in this field into the clinical setting highlighted mixed results with benefits ranging from absent to transient or, at most, marginal [9, 10]. A part of the delay in the therapeutic advancement in the field of stem cells and cardiac regeneration is caused by the fact that little is still known about the mechanisms to increase stem cell survival after in vivo transplantation and to efficiently induce its transdifferentiation into mature cardiomyocytes. These mechanisms need to be urgently elucidated in future investigations. In addition, the standardization of the procedures for isolation, purification, manipulation, and transplantation of human stem cells used for therapeutic purposes needs to be implemented, despite the fact that the standards of safety and quality for stem cell therapeutic uses are currently defined in the Good Manufacturing Practice (GMP) guidelines (see Eudralex EU guidelines for Good Manufacturing Practice for Medicinal Products for Human and Veterinary Use) [11, 12]. Nonetheless, full compliance with GMP is a mandatory aspect of stem cell-tissue engineering and manufacturing.

The improvement of translational research, however, cannot represent alone the solution for developing new strategies for the cure of cardiovascular diseases. Together with translational research, it would be highly important to implement also clinical and outcome research. These scientific research branches aim to further expand the current knowledge of the prevalence, incidence, impact, and management of cardiovascular abnormal conditions in selected or real-world patients. This would help to identify shortfalls in practice and to develop strategies to improve care. In particular, outcome research is planned to continuously provide new insights into the therapeutic interventions working best for specific types of patients and under specific circumstances.

Given these premises, this special issue aimed at integrating expertise from different disciplines toward the same objective: a deeper understanding of the mechanisms underlying cardiovascular diseases as well as the development of new therapeutic strategies to prevent or treat cardiovascular diseases. In our opinion the result was notable. Among the accepted manuscripts, some studies developed new methods enhancing the cardiovascular transdifferentiation of stem cells or standardized the procedures for the isolation of bone marrow-derived cellular subtypes for the treatment of refractory ischemia. Other manuscripts dealt with the molecular mechanisms underlying ischemia/reperfusion damage or heart failure with preserved ejection fraction. The biomarkers associated with resistant hypertension or aortic aneurism rupture were also studied. In addition, some epidemiologic studies provided new insights into the factors associated with coronary artery disease or with a worse cardiovascular outcome. Finally, the genetic basis of metabolically unhealthy obesity was also investigated.

The editors really hope that the scientific contributions accepted for this special issue may contribute in some extent to the advance of the current knowledge of the pathophysiology, prognosis, and management of cardiovascular diseases.

*Giacomo Frati* *Umberto Benedetto* *Giuseppe Biondi-Zoccai* *Sebastiano Sciarretta*
